# Genomic sequence and copy number evolution during hybrid crop development in sunflowers

**DOI:** 10.1111/eva.12603

**Published:** 2018-02-20

**Authors:** Gregory L. Owens, Gregory J. Baute, Sariel Hubner, Loren H. Rieseberg

**Affiliations:** ^1^ Department of Botany and Biodiversity Research Centre University of British Columbia Vancouver BC Canada; ^2^ Department of Biotechnology Tel‐Hai Academic College Upper Galilee Israel; ^3^ MIGAL ‐ Galilee Research Institute Kiryat Shmona Israel

**Keywords:** copy number variation, genomics, heterosis, hybridization, postdomestication diversification, presence/absence variation

## Abstract

Hybrid crops, an important part of modern agriculture, rely on the development of male and female heterotic gene pools. In sunflowers, heterotic gene pools were developed through the use of crop‐wild relatives to produce cytoplasmic male sterile female and branching, fertility restoring male lines. Here, we use genomic data from a diversity panel of male, female, and open‐pollinated lines to explore the genetic changes brought during modern improvement. We find the male lines have diverged most from their open‐pollinated progenitors and that genetic differentiation is concentrated in chromosomes, 8, 10 and 13, due to introgressions from wild relatives. Ancestral variation from open‐pollinated varieties almost universally evolved in parallel for both male and female lines suggesting little or no selection for heterotic overdominance. Furthermore, we show that gene content differs between the male and female lines and that differentiation in gene content is concentrated in high F_ST_ regions. This means that the introgressions that brought branching and fertility restoration to the male lines, brought with them different gene content from the ancestral haplotypes, including the removal of some genes. Although we find no evidence that gene complementation genomewide is responsible for heterosis between male and female lines, several of the genes that are largely absent in either the male or female lines are associated with pathogen defense, suggesting complementation may be functionally relevant for crop breeders.

## 1 INTRODUCTION

The process of crop domestication—in which a wild plant is modified by a combination of conscious and unconscious selection into something more amenable to human use—is frequently distinguished from crop differentiation and improvement. The latter two processes are characterized by conscious selection for traits or varieties for particular locations or specific uses. In sunflower, for example, domestication primarily involved changes in plant architecture (from many lateral branches subtended by numerous small flowering heads to an unbranched stem topped by a single large flowering head), increased achene (one‐seeded fruit) size, and loss of seed dormancy, shattering, and self‐incompatibility (Burke, Tang, Knapp, & Rieseberg, 2002). Subsequent differentiation and improvement resulted in two main cultivar groups: oil versus confectionary varieties, as well as other minor differences. For example, the Hopi Dye sunflower was selected for its purple black achenes, which were used by the Hopi as a natural dye source for coloring baskets. Likewise, numerous horticultural varieties have been developed, including the famous Van Gogh sunflower, which results from altered expression of a CYCLOIDEA‐like gene (Chapman et al., 2012).

In the early 1970s, sunflower breeders developed “male” (R or restorer lines) and “female” (B or maintainer lines) lines, with the goal of maximizing heterosis or hybrid vigor, which is a critical component sunflower production. As with other hybrid crops, yield gains from heterosis are considerable in sunflower (up to 30%; Fick & Swallers, 1972). Over the past half century, sunflower breeders have further developed heterotic groups to maximize heterosis. These heterotic groups are composed of individual lines that, when crossed within a group, do not generate large heterotic effects, but when crossed between groups yield pronounced hybrid vigor.

Breeders rely on these heterotic gene pools to predictably produce high yielding hybrids. As with other crops the boost in yield from heterosis, combined with legal and biological protection provided by F1 seeds (i.e., saved F2 seed will segregate), has resulted in a large hybrid seed industry. Sunflower is one of the most valuable hybrid crops, with a global seed market of circa 1 billion USD annually. In addition, maize, tomato, cotton, sorghum, and others are all primarily grown as hybrid crops. It is noteworthy that heterosis may not be exploitable in all crops. For example, despite considerable investment into its development, hybrid wheat may not experience commercial success in the near future because of the absence of an efficient hybrid seed production system, as well as the genetic architecture of key traits (Whitford et al., 2013). Given potential yield increases from heterosis, and the associated success of the hybrid seed industry, there is considerable incentive to investigate the genomic consequences of heterotic gene pool development, as well as the mechanisms of heterosis.

Three general genetic models have been put forward to account for heterosis: dominance, overdominance, and epistasis. In the dominance model of heterosis, the enhanced performance of hybrids is thought to result from genetic complementation (i.e., the masking of deleterious recessive alleles from one parent by dominant alleles from the other parent). The overdominance model posits that increased hybrid vigor is due to favorable interactions of alleles from different lineages at a single locus (i.e., that heterozygotes are more fit than homozygotes). The third mechanism, epistasis, presumes that the superior performance of hybrids results from beneficial interactions of parental alleles at different loci. In addition to considerable empirical support for each of these genetic models (see below), an increasing number of studies have characterized the underlying molecular mechanisms (Chen, 2013), which are largely consistent with one or more of the genetic models described above. Molecular processes commonly associated with heterosis include changes in gene expression (Krieger, Lippman, & Zamir, 2010; Swanson‐Wagner et al., 2006), protein metabolism (Goff, 2011), copy number and gene presence–absence variation (Lai et al., 2010; Springer et al., 2009; Swanson‐Wagner et al., 2010), and epigenetic modification of key regulatory genes (Chen, 2013). At the sequence level, overdominant selection should lead to different alleles fixing in heterotic groups; in contrast, dominant selection may lead to the same allele fixing. Epistasis, since it involves the interactions of multiple loci across the genome, makes no simple predictions. In this paper, we focus on the first two models because we lack QTL data to identify the third.

While experimental data have been found to support each of the genetic models for heterosis, determining the relative importance of these different explanations has been much more challenging. Early genetic studies of heterosis often relied on QTL mapping, but detection of underlying QTLs can be affected by statistical and technical considerations (Schnable & Springer, 2013). For example, unless carefully designed, QTL studies often have limited power to detect epistasis. An additional issue is a phenomenon called pseudo‐overdominance, which can make it difficult to distinguish between the dominance and overdominance models (Schnable & Springer, 2013). Pseudo‐overdominance is caused by tight linkage between a pair of dominant alleles in repulsion phase, giving the appearance of overdominance. Limited recombination in cultivated populations could mean that pseudo‐overdominance plays a big role in heterosis, as has been suggested in maize (Gore et al., 2009). Most QTL studies investigating heterosis have reported that a large number of small effect loci are involved (Schnable & Springer, 2013). However, several studies have found a single gene with large overdominance effects, for example, in tomato (Krieger et al., 2010) and in *Arabidopsis* (Ni et al., 2009).

Before hybrid sunflower cultivars were developed in the 1970s, all production employed open‐pollinated varieties (OPVs). These OPVs were not subject to severe inbreeding, as are modern inbreds, but instead were maintained as small populations. From these OPVs, the male and female gene pools were developed (Korell, Mösges, & Friedt, 1992). The critical attribute that divided these groups was the presence of cytoplasmic male sterility (CMS) in the female (maintainer) lines and complementary restorer alleles in the male (restorer) lines. Additionally, the restorer lines have had branching reintroduced. Both CMS (Leclercq, 1969) and the restorer and branching alleles were brought into cultivated sunflowers from wild relatives (Fick, Kinman, & Zimmer, 1975; Kinman, 1970). Breeders have and will continue to select for heterosis in the development of these gene pools. Although much phenotypic evaluation of new potential inbred lines is done on the inbred lines themselves, test crossing is usually a critical component of line selection, and combining ability is a top priority in inbred line release and use. Thus, breeders have selected for heterosis in these sunflower gene pools for several decades.

Here, we use whole‐genome sequence (WGS) data from a diverse panel of OPV, restorer line, and maintainer line varieties to investigate the genomic impact of this selection. The profile of selection on these genomes may provide clues regarding (i) the genetic consequences of heterotic line development and (ii) the mechanisms responsible for heterosis, as well as (iii) useful information for the continued improvement of sunflower.

## 2 METHODS

### 2.1 Sampling and genotyping

To investigate the impact of selection during the creation of the heterotic gene pools, we leveraged sequence information developed for a public sunflower association mapping population. This population was developed as a community effort and attempts to capture as much of the diversity in cultivated sunflower as possible (Mandel et al., 2013). Lines were selected from numerous gene pools, including landraces, OPVs, and modern high oil lines and where necessary, accessions were advanced via single‐seed descent for one or two generations to minimize residual heterozygosity. Most relevant to this study are a large sampling of restorer (96 samples) and maintainer varieties (127) and OPVs (9) from which they have been derived. This whole population was sequenced to a target of 5–20× coverage on the Illumina platform (Table S1), although aligned median read depth ranged from 1.6 to 19 (median 4.5) (Hubner et al., 2018).

All sequence data were trimmed for poor‐quality reads and adapter sequence using Trimmomatic (v.0.36), aligned to the *Helianthus annuus* XRQ reference (Badouin et al., 2017) using an aligner developed by SAP SE (Waldorf, Germany), which is optimized to use available main memory for faster indexing, minimize the cache miss ratio to improve performance, and optimize parallel code execution (Bolger, Lohse, & Usadel, 2014). Following this, PCR duplicates were removed using Picardtools (v2.5) and variants were called collectively using FreeBayes (v1.0.0) (Garrison & Marth, 2012). The raw variants were then thoroughly filtered to produce a set of high confidence biallelic SNPs with vcftools and the following parameters: QUAL > 30, observed heterozygosity < 0.3, depth for individual genotype > 1, minimum average depth > 1, max average depth < 16, no indels, two alleles, genotype known in the reference genome, no strand bias (SAR & SAF > 0, RPL & RPR > 1), minor allele frequency > 0.05, and missing data < 0.3.

### 2.2 Genetic differentiation between groups

We calculated F_ST_ between open‐pollinated (OPV), male (restorer) and female (maintainer) samples using a custom Perl script and averaged scores across 500 kb windows by summing the numerator and denominator of F_ST_ for all markers in the window (Weir & Cockerham, 1984). We required markers to be called in >1 sample per group and have a minor allele frequency > 0.05 and observed heterozygosity <0.6. Additionally, we ran a PCA on all samples with a random (~1%) subset of sites (18,344 total) using the SNPrelate package in R (Zheng et al., 2012). Variants were trimmed for LD (*r* ^2^ > .2) before PCA.

### 2.3 Gene copy number quantification

To identify gene copy number variants, we first aligned all trimmed data to the *H. annuus* XRQ genome using NextGenMap (v0.5.3) (Sedlazeck, Rescheneder, & Von Haeseler, 2013). This aligner is designed to align sequences more highly divergent from the reference sequence than other aligners, so it should be less prone to not aligning divergent alleles in genes. As before, PCR duplicates were flagged using Picardtools.

There are several published methods for quantifying gene copy number using sequencing data (Duan, Zhang, Deng, & Wang, 2013; Krumm et al., 2012; Yoon, Xuan, Makarov, Ye, & Sebat, 2009). Most are designed for human genetics and require high depth (>20×). In this dataset, we had lower average depth due to the large sunflower genome (3.6 Gb). Additionally, the sunflower genome is exceptionally repetitive and many of the genes have little to no coverage when mapping quality filters are used as recommended by most programs. With this in mind, we employed a simple and conservative approach to call copy number variants. We first quantified average per base read depth for the full sequence of each gene in the XRQ genome without filtering for mapping quality. Then for each sample, we calculated the median gene depth and classified each gene into one of three categories: absent (zero reads), amplified (>3*median reads), or present (>zero reads, ≤3*median reads). With this classification, we may be under‐calling the effective number of missing genes because partial deletions would be counted as present, but we are less likely to call genes as missing simply due to stochastic variation in read depth. We also will only detect gene deletions when they are homozygous.

Although we detected differences in the number of absent genes between our groups, our simplistic way of calling gene presence/absence variation (PAV) may be prone to false positives for samples with fewer reads so we explored the relationship between median depth and the number of missing genes for a sample. To do this, we ran a nested linear regression without interaction effects to test sequentially for the effect of median depth and group identity on the number of absent genes. We also ran a permutation test, where we randomly rearranged group identity and recalculated the number of genes that have different frequencies of amplified or absent genes. This allows us to see if the amount of differentiation we see between maintainer and restorer lines is more than expected with just measurement noise.

To explore the link between introgression and gene loss, we used HA412HO, which previously had its introgressions finely mapped (Badouin et al., 2017). We compared the proportion of genes that are absent in and outside of introgressed regions. For this analysis, we captured a single introgression value for each gene, which represented the window overlapping the gene with the highest proportion of introgressed ancestry. To classify a region as introgressed, we required it to have >0.1 non‐*H. annuus* landrace ancestry. A chi‐squared test was used to check if there was a difference in the proportion of missing genes within and outside of introgressed regions.

Lastly, although the restorer of fertility locus *Rf1* has been mapped to chromosome 13, we still do not know the genetic identity of the locus. To identify candidate genes, we queried the XRQ genome for “pentatricopeptide” labeled genes, which have frequently been shown to underlie fertility restoration in other crops, and extracted genes in high F_ST_ (>0.2) 100‐kb windows on chromosome 13.

### 2.4 Exploring the causes of heterosis

One way that heterosis can occur is through overdominance, in which growth rate, yield, or fitness is highest when a given locus is heterozygous. Signatures of selection from overdominance are difficult to detect because directional selection in a single group will also increase the heterozygosity of crosses between groups. For example, an allele that increases yield for single‐headed sunflowers may be selected for in maintainer but not restorer lines. Crosses between maintainer and restorer lines will have high heterozygosity at this locus, but it will not be due to any selection on overdominance. Thus, looking at heterozygosity itself is not always informative.

**Figure 1:**
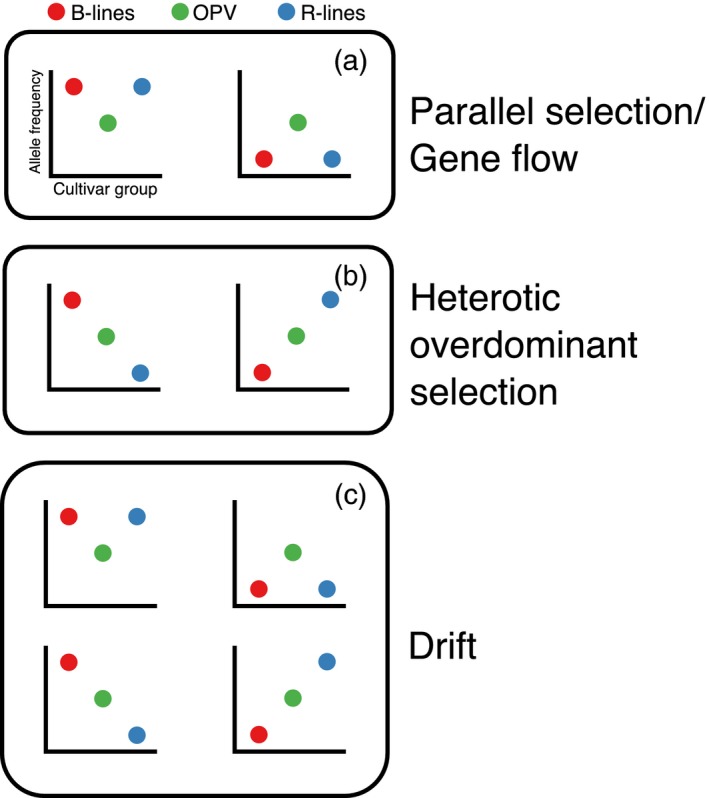
A model of how different forces could modify allele frequencies in the cultivar groups. Maintainer = B‐line, restorer = R‐line, open‐pollinated variety = OPV‐line. (a) Parallel selection or gene flow will cause shifts in allele frequency in the same direction. (b) Heterotic overdominant selection will cause male and female lines to shift to different alleles. (c) Drift can cause shifts in any direction

To explicitly look for signs of selection on overdominant heterosis, we searched for loci where the open‐pollinated variety was polymorphic and the restorer and maintainer lines have shifted in allele frequency toward opposite alleles. For overdominant loci, selection on heterosis may drive alternate alleles to fix in the two heterotic groups. In contrast, parallel directional selection on additive or dominant general improvement traits (i.e., disease resistance) will result in parallel shifts in both heterotic groups. Gene flow between heterotic groups during breeding will homogenize allele frequencies and produce results that are similar to those predicted by parallel selection. Lastly, drift could shift allele frequencies in either direction and produce equal rates of parallel and nonparallel shifts (Figure 1).

We selected sites where the OPV had an alternate allele frequency between 0.25 and 0.75. This ensures that there is ancestral variation that can be sorted in the heterotic lines. We then selected sites where both the maintainer and restorer lines had shifted their allele frequency by atleast 0.2 as compared to the OPV. We then categorized the loci by whether both restorer and maintainer lines shifted their allele frequency in the same direction or not.

Complementation of missing genes could also play an important role in heterosis. To explore this, we simulated crosses between random individuals between and within cultivar groups. For each cross, we counted the number of genes where both individuals were missing the same gene. We then asked if there were fewer missing genes in the maintainer to restorer line cross, as expected if complementation drives heterosis.

## 3 RESULTS

### 3.1 Genetic differentiation

**Figure 2:**
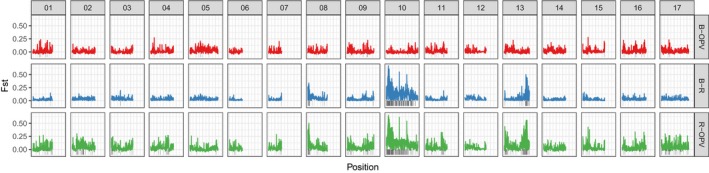
F_ST_ between maintainer lines and open‐pollinated varieties (OPV) (red), maintainer and restorer lines (blue), and restorer lines and OPV (green). Values are plotted for 500 kb nonoverlapping windows. The gray bars under the F_ST_ scores highlight regions with highest 5% of windows for each comparison

We find that F_ST_ was fairly low overall, but the highest F_ST_ comparisons always included the restorer lines (Table 1). For these comparisons, there was significant variation in F_ST_ values and the highest values were concentrated in chromosome 10 and, to a less extent, chromosomes 8 and 13 (Figure 2). The patterns of divergence are similar to those reported previously between restorer and maintainer lines based on a less complete reference sequence (Hubner et al., 2018). However, differentiation relative to OPVs was not reported in the previous study, so the directionality of change could not be inferred.

**Table 1:** Genome‐wide F_ST_ between cultivar groups

F_ST_	Maintainer	Restorer	OPV
Maintainer	NA		
Restorer	0.045	NA	
OPV	0.014	0.054	NA

OPV, open‐pollinated varieties.

**Figure 3:**
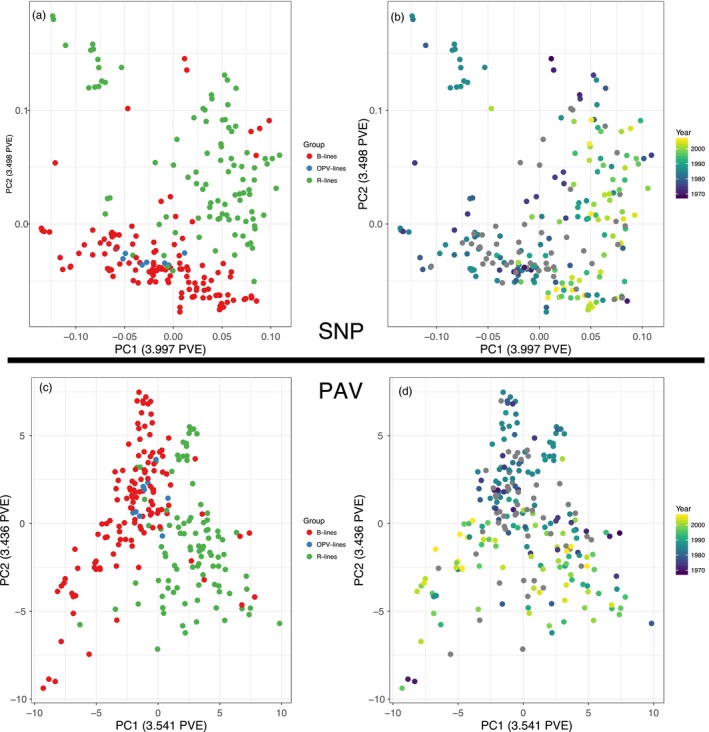
Principal component analysis of SNP (a,b) and presence–absence variation (c,d). SNP analysis was run in SNPrelate and was pruned for linkage. Samples are color‐coded by cultivar group (a,c) or the year the line was released (b,d). Maintainer = B‐line, restorer = R‐line, open‐pollinated variety = OPV‐line

Consistent with the F_ST_ scores, principal component analysis separated restorer and maintainer lines in the second axes of variation (Figure 3a,b). Open‐pollinated varieties grouped with maintainer line samples.

To help identify the restorer locus *Rf1* found in male lines, we searched the genome for candidate genes. We found ten genes in high F_ST_ regions labeled as “pentatricopeptide.” Due to the high stringency in which we filtered SNPs, we had zero SNPs in most genes, so we are unable to narrow this window to specific genes (Table S2).

### 3.2 Presence/absence and copy number variation

**Figure 4:**
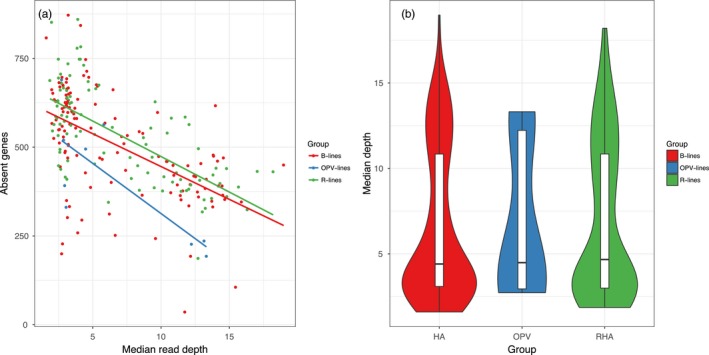
Exploring the possible effect of read depth on absent gene counts. (a) The relationship between median read depth and the number of absent genes. Samples are color‐coded by cultivar group, and a linear regression is plotted for each. (b) The mean read depth distribution for each cultivar group. There is no significant difference between the groups. Maintainer = B‐line, restorer = R‐line, open‐pollinated variety = OPV‐line

We detected 6,487 genes with presence–absence variation. Of those, 4,941 were absent in more than one sample, giving us more confidence that they present real variation and not measurement error. We found one gene, HanXRQChr08g0211631, that was not present in any samples. The number of missing genes per sample varied from 872 (HA268) to 36 (XRQ, which was resequenced as part of the sunflower association mapping population) (Figure S1a,b). We find a strong effect of median depth for the number of absent genes (i.e., lower depth means more absent genes) (Figure 4a). While controlling for median depth, we found significant differences in the number of absent genes between different groups (*F*(3, 228) = 49.09, *p *<* *2.2e^−16^, *R* ^2^ = .3845). This showed that restorer lines had the most missing genes and OPV the least. Additionally, we find no differences in mean depth between different groups of samples (*F*(2, 229) = 0.104, *p *=* *.9, *R* ^2^ = .0009; Figure 4b).

For amplified genes, we found 4,402 genes with that were amplified in at least one sample, including 49 amplified in all samples (Figure S1c). Individual samples had between 145 and 1,491 amplified genes (Figure S1d).

**Figure 5:**
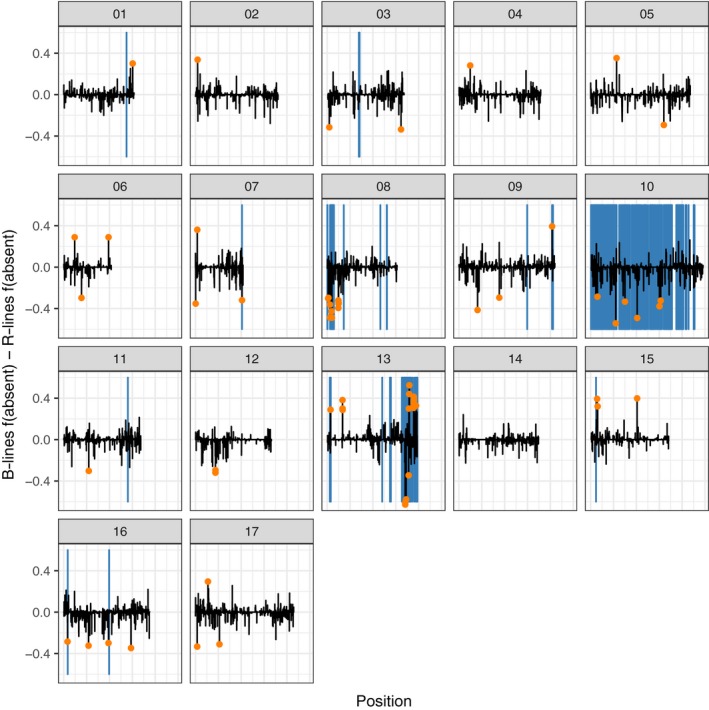
Difference in frequency of absent genes between maintainer (B) and restorer (R) lines. Negative values represent genes that are more often absent in restorer lines; positive values are genes that are more often absent in maintainer lines. Orange dots highlight the top 1% of differentiated genes. Blue bars highlight the top 5% F_ST_ windows

We explored gene PAV by running a principal component analysis and found that the major axis of variation separated maintainer and restorer line samples (Figure 3c,d). To determine the genes differentiating these groups, we calculated the difference in frequencies of missing genes and plotted them across the genome. We find that these differentiated genes are concentrated in regions of high F_ST_ in chromosomes 8, 10, and 13 (Figure 5, Figures S2 and S3; Table S3). The number of differentiated genes, for both absent and amplified, is far higher than any permutation, showing that this represents real genetic variation (Figure S4).

**Figure 6:**
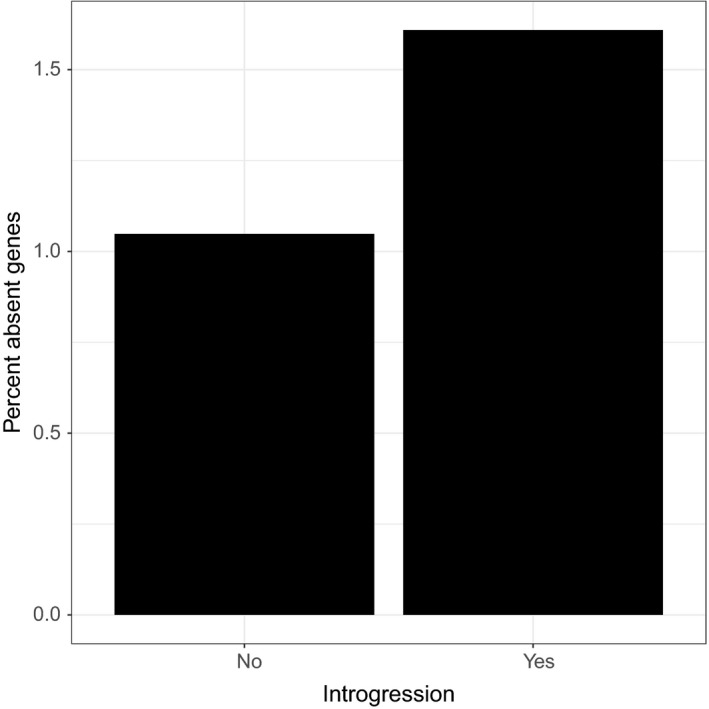
The percent absent genes for HA412HO inside and outside of probable introgressed regions. Introgressed regions were identified using Structure linkage model (Badouin et al., 2017)

Since gene losses cluster in putative introgressed regions, we explored this further in the HA412HO genome, which has had its genome scanned for introgressions. We find that in the HA412HO genome, absent genes are 50% more likely to occur in introgressed regions than in nonintrogressed regions (chi‐squared test, *p*‐value = .0038). In total, 1.5% of genes are absent in introgressed regions and only 1% in nonintrogressed regions (Figure 6).

### 3.3 Heterosis

**Figure 7:**
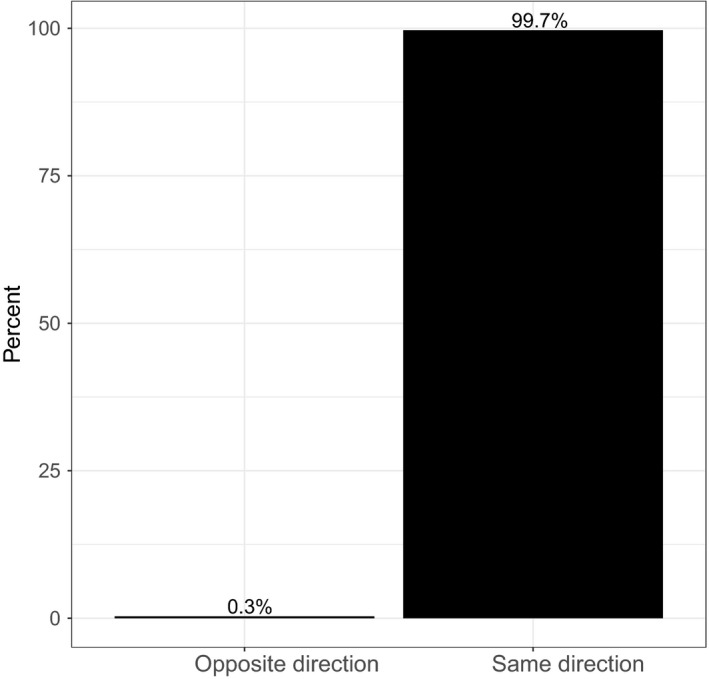
The fate of ancestral variation in open‐pollinated varieties (OPV) where both maintainer and restorer lines have diverged from the ancestral frequency. Loci either both shift in the same direction or they shift in opposite directions

We find that when both restorer and maintainer lines have changed their allele frequency during improvement, they almost always have shifted in the same direction. This occurs in 99.7% of sites we observed (Figure 7). However, there are areas of the genome where opposite shifts in allele frequency are more common, particularly on chromosome 10 from 193 to 203 Mb (Figure S5).

As heterosis may be caused by the complementation of absent genes, we simulated crosses by permuted pairs of samples and asked how often the same gene was absent in both. We found that, although restorer to maintainer line hybrids had fewer absent genes than crossing either within their heterotic group, theoretical hybrids involving OPV‐lines had the fewest missing genes (Figure S6).

## 4 DISCUSSION

In this paper, we have investigated the genomic changes associated with one of the most recent innovations in plant breeding, the development of hybrid crops. Using sunflower as an example, we find that much of the genomic change is concentrated in the male‐fertile restorer lines and is a consequence of introgressions from wild sunflower relatives. Importantly, these introgression events have modified gene content, including removing known genes, with potentially important functional implications. An even bigger surprise is that the ancestral polymorphisms found in the OPV ancestors of the male and female lines seem to have shifted allele frequency in the same way in the male and female lines. Together, these results imply that overall, the heterotic groups in sunflowers were not selected for heterotic overdominance. Below we describe caveats associated with these observations, offer potential explanations for our results, and discuss their broader implications for plant breeding and hybrid crop development.

### 4.1 Challenges in quantifying gene presence/absence variation:

There are both biological and methodological factors that may bias which genes are identified as being absent in our analysis. First, due to genome size of sunflower, we have not sequenced each sample exhaustively and some genes scored as absent are present, but we have failed to detect them due to stochastic variation in read placement as evidenced by the relationship between the number of missing genes and the median read depth (Figure 4). This is exemplified by the fact that our XRQ sample is missing 36 genes. The reference genome is from the XRQ inbred line, so all genes in the reference genome should be present in our XRQ sample, although it should be mentioned that the genome was sequenced using PacBio sequencing, and thus may not have the same biases. These might also be in areas of the genome that are more difficult to sequence, for example, areas with high GC content (Botero‐Castro, Figuet, Tilak, Nabholz, & Galtier, 2017). The samples with the highest number of absent genes (>800) all have <5 median read depth. Although this relationship seems to hold at low read depth, above 10 median read depth the relationship is much weaker or nonexistent suggesting that additional sequencing and re‐analysis will not detect all missing genes and that some of the variation is real. We do want to emphasize that while low read depth can lead to failures to detect genes that are present, there is no difference between the median read depths of our different groups, so this is not driving the patterns we observe.

Another bias is the length of the gene. Shorter genes are more likely to be missed entirely during sequencing, but also, shorter genes are more likely to be removed entirely by structural variation. In our data, we find that the genes with presence/absence variation are strongly biased toward shorter genes but we cannot disentangle these two biases (Figure S7). It is distinctly possible that longer genes have presence/absence variation that does not involve the entire sequence but is functionally equivalent and was not detected in our survey.

It is theoretically possible that genes identified as absent are present but are so different from the reference sequence that reads do not align. We consider these cases as functionally absent as they would need to be substantially diverged to not align. With all these possible biases, we emphasize that there is real genetic variation in gene presence/absence. This is supported by several results. First, the difference in PAV frequency between the restorer and maintainer lines is far higher than any permutation of sample identity. Second, the first axis of PAV separates restorer and maintainer lines. Lastly, the sample with the fewest absent genes is XRQ, the same line that the reference genome is based on, and the sample that should have the fewest absent genes.

### 4.2 Introgression brings gene loss in restorer lines

During the development of the restorer lines, branching was intentionally reintroduced by crosses with wild sunflower collections from Texas, *H. annuus sp. texanus* (Fick et al., 1975; Kinman, 1970; Korell et al., 1992). QTLs for branching map to chromosomes 8 and 10 and colocalize with identified introgression blocks from the purported donor taxon (Baute, Kane, Grassa, Lai, & Rieseberg, 2015; Mandel et al., 2013). The other major functional trait in the restorer lines is male fertility, which is restored by the *Rf1* locus, which maps to chromosome 13, and likely also came from a wild donor (Horn, Kusterer, Lazarescu, Prüfe, & Friedt, 2003; Yue, Vick, Cai, & Hu, 2010). While these introgressions were created for specific phenotypic traits, they also shaped the gene content of the resulting lines.

We find that the restorer lines have disproportionately more gene losses and gene amplifications than the OPV‐ or maintainer lines. This is partially a consequence that the reference used, XRQ, is a maintainer line sunflower. This means that genes specific to the maintainer line would be present in the reference genome and picked up by this study, but genes specific to the restorer lines would not be in the reference genome and would be ignored. A similar argument would hold for copy number variants underlying the amplified genes we identify. The OPV samples, which are the progenitors of both the maintainer and restorer lines, are more similar to the maintainer lines in both sequence and copy number variation, so the divergence in restorer lines is likely a derived characteristic.

The largest difference in PAV between restorer and maintainer lines is concentrated in the introgressed chromosomes 8, 10, and 13 (Figure 5). Thus, we propose that introgression is removing genes during the breeding process. To confirm this, we examined cultivar line HA412HO, which has had introgressed regions in its genome previously mapped (Badouin et al., 2017). As predicted, we find that absent genes are much more common in the introgressed regions (Figure 6). Of course, these introgressed regions are likely also bringing in new genes but we are not detecting those because we rely on the XRQ reference gene complement and not a *de novo* assembly approach. Thus, we are not saying that introgression reduces total genes, but that it simply removes some reference genes. Introgression presumably also adds new genes, although we do not address that here.

Although evidence of different pan‐genome gene content between inbred lines in sunflowers was recently shown (Hubner et al., 2018), the role of introgression in gene loss has not been previously appreciated. Missing genes may play a role in linkage drag, which is a concern to breeders when wild relatives are employed for improvement (Curwen‐McAdams & Jones, 2017). Linkage drag refers to the often maladaptive impact of genetic material that is introduced into cultivated lines along with the beneficial trait that is targeted by selection. While such maladaptive effects are typically assumed to result from the introduction of deleterious genes, they could also be due to the absence of key genes, which our results suggest might be a common consequence of introgression.

### 4.3 Parallel evolution and a lack of evidence for heterosis through overdominance

In maize, enormous work has been done dissecting the genetic basis of heterosis, including precise measurements of heterotic potential (i.e., combining ability) using dozens of lines (e.g., Riedelsheimer et al., 2012). In sunflowers, comprehensive studies of heterotic potential have not been conducted, or at least such studies are not publically available (although see Cheres, Miller, Crane, & Knapp, 2000). Lacking this information, we looked for patterns suggesting how selection is acting. We find that in an overwhelming majority of cases (99.7%!), maintainer and restorer lines are sorting ancestral variation in concert. While both gene flow between male and female lines and parallel selection could cause this pattern, pedigree records imply that the former is rare. A third possibility is that both the male and female lines originated from a restricted and genetically homogenous group of OPVs.

Although we emphasize that parallel sorting of ancestral alleles is almost universal, this is not to say that the maintainer and restorer lines are genetically identical; for much (~79%) of the ancestral variation segregating within OPV, maintainer and restorer lines are similar to OPV or only a single line has diverged. The loci where both maintainer and restorer lines have diverged, which we examined, represent only 21% of the total segregating variation in OPV. Maintainer and restorer lines also differ from each other at alleles rare or not present in OPV. Additionally, both the maintainer and restorer lines have had numerous wild introgressions which in many, or most, cases are unique to one group or the other.

Genome‐wide, we find very little evidence for overdominant heterotic selection. As the sorting of alternative ancestral alleles in male versus female lines could be caused by drift alone, and the ratio is far below the 50% predicted by drift, we cannot confidently determine if selection for overdominant heterotic selection is acting at all. That being said, there are genomic regions where the sorting of alternate alleles is more common (Figure S5). This occurs mainly in high F_ST_ windows (as expected because the sorting of alternate increases F_ST_), but is particularly high in a 10‐Mb region on the end of chromosome 10. This region has elevated F_ST_ but is not particularly high for the chromosome; the highest peaks occur earlier. However, as this area of the chromosome is responsible for a recessive branching phenotype, which has been constructed for F1 seed production, it does not represent a good candidate for overdominant heterosis.

Both copy number variation and PAV have been implicated for heterosis in maize (Lai et al., 2010; Springer et al., 2009; Swanson‐Wagner et al., 2010). Lai et al. identified 296 genes present in the B73 reference genome but absent in one or more inbred lines, and 570 present in the tested inbred lines and absent in B73. Furthermore, heterotic groups contained largely different sets of missing genes suggesting the possibility of heterosis occurring through complementation of missing genes. Heterotic groups are genetically distinct, so divergent sets of missing genes are expected simply due to population structure regardless of their effect on heterosis or lack thereof. Stronger evidence of PAV affecting heterosis was found by Jin et al., who correlated heterosis and ePAV (expressed presence–absence variation) for a set of maize crosses (Jin et al., 2016). Since this study used expression data, it is unknown how much of the heterotic effect was due to differences in gene expression regulation versus actual gene absence.

In our dataset, although maintainer and restorer lines have different complements of missing genes, complementation of missing genes is highest in simulated crosses with OPVs, because OPVs have the fewest number of missing genes overall (Figure 4a). That being said, it is important to remember that with our approach, gene absence can only be scored when homozygous. The OPV‐lines are maintained as populations and are heterozygous at many more variable sites, whereas the maintainer and restorer lines are inbred and highly homozygous (Figure S8). Thus, the OPV may not actually have fewer missing genes, but just fewer homozygous missing genes. This means that real crosses may not actually have fewer missing genes in crosses involving OPV as heterozygous missing genes will be revealed.

Although we do not find an aggregate effect of PAV for heterosis, it is distinctly possible that individual PAV plays a role. Two of the top eleven genes with PAV differentiating restorer and maintainer lines are purported defense genes. HanXRQChr13g0421621 is a homologue of RPP13, which controls downy mildew resistance in *A. thaliana* (Bittner‐Eddy, Crute, Holub, & Beynon, 2000). This gene is present in most of the restorer line samples and absent from a majority of OPV and maintainer lines and occurs in a genomic region previously shown to be derived via introgression with a wild species (Baute et al., 2015). The gene also colocalizes within the best supported downy mildew QTL reported by Hubner et al. (2018). The correlation of gene presence and introgression may suggest that presence–absence variation is segregating within both cultivars and wild relatives. In this case, the “absent” allele is more common in cultivated sunflower (although present in the XRQ reference genome) while the wild relative donor had the “present” allele. In a reciprocal fashion, HanXRQChr08g0209581 encodes a TMV resistance N‐like gene, which is associated with virus resistance in tobacco, and others (Guo et al., 2017; Stange, Matus, Elorza, & Arce‐Johnson, 2004). This gene is absent in the restorer lines, but present in the maintainer and OPV‐lines, possibly suggesting introgression removed it.

Overall, our analyses support the role of selection on dominant and simple additive variants during heterotic group development and fail to find support for overdominant selection (with caveats). Ancestral variation was overwhelmingly sorted in parallel in maintainers and restorers, this could be explained by a majority of selected traits being additive or recessive in nature and being selected upon during inbred development in parallel in both groups or by advancing lines, which create hybrids that are homozygous at these loci. Dominance likely plays an important role in addressing the PAV introduced from wild introgressions. Dominant loci are likely favored by breeders when using wild relatives because they only need to be introgressed into one gene pool and heterozygosity may also reduce the likelihood of encountering linkage drag. We have not evaluated the role of epistasis so we cannot say how large of a role it plays in sunflower heterosis. Future work could use QTL analysis to identify epistatic loci involved in heterosis (Yu et al., 1997).

### 4.4 Identity of the *Rf1* restorer loci

Male fertility restoring loci are essential for the commercial viability of most hybrid crops. In sunflowers, the most common form of male sterility is interspecifically derived from *H. petiolaris* mitochondria, while fertility restoration is from the restorer loci *Rf1* and *Rf2* (Leclercq, 1969). While *Rf2* is present in almost all inbred lines, it is the presence of *Rf1* that can restore male fertility in lines with this cytoplasm (Leclercq, 1969). Genetic mapping has localized *Rf1* to chromosome 13 but the genetic identity is unknown (Horn et al., 2003). If this was known, it would allow for more closely linked markers to be created or perhaps for the creation of novel restorer alleles using transgenics or gene editing.

Here, we focus our search on pentatricopeptide repeat (PPR) proteins because they have been repeatedly identified as restorer loci in other species (Chen & Liu, 2014). We find 10 candidate genes that are in high F_ST_ windows colocalizing with the *Rf1* genetic map location. Unfortunately, we are unable to narrow this window due to the stringency of our filtering, but future fine mapping should target these genes.

Interestingly, we find two candidate genes that are differentiated in gene copy. Both HanXRQChr08g0207431 and HanXRQChr13g0419821 are in much higher copy number in the restorer lines than the maintainer lines. HanXRQChr08g0207431 encodes a putative PPR gene that is mostly single copy in maintainer and OPV but has approximately four copies in restorer samples. Although this gene maps to chromosome 8 and not chromosome 13, as expected for *Rf1*, the increased copy number may reside elsewhere in the genome. HanXRQChr13g0419821 encodes an aldehyde dehydrogenase gene. In maize, the restorer gene *rf2* is an aldehyde dehydrogenase and fertility restoration functions through the gain of aldehyde dehydrogenase function (Liu, Cui, Horner, Weiner, & Schnable, 2001). It is possible that the amplification of HanXRQChr13g0419821 (found at ~10× more copies in restorer samples) increases aldehyde dehydrogenase activity and functions in the same manner.

## 5 CONCLUSIONS

The breeding of heterotic gene pools has been critically important for the success of sunflower as a modern crop, and understanding the genomic basis of this heterosis is important for continued improvement. Our survey of genomic variation in the heterotic lines has two major findings. First, the ancestral variation in OPV was overwhelming sorted in parallel between both heterotic groups. This suggests that there may be underexploited variation within OPV and mining older lines for useful traits could be fruitful. Another possibility is that the haplotypes selected in parallel in both heterotic groups contain desirable recessive traits, in which case breeders may seek new haplotypes, possibly from wild donors, that still produce this trait but bring in additional useful variation. Our second finding is that introgression during breeding can result in the loss of genes. This may be one cause of linkage drag that can be frequently observed when breeding with wild germplasm. The preferred approach to address this is to use a dominant allele for a given trait; however, this is not always possible. When only recessive or additive alleles are available, using haplotypes which are as unrelated as possible in the restorer and maintainer gene polls may help avoid any linkage drag issues. These alleles may be found in different donors or generated from a single donor using different genetic backgrounds and targeted recombination to reduce the size of the introgressed region.

## CONFLICT OF INTEREST

The authors declare no conflicts of interest.

## DATA AVAILABILITY

All scripts used in this analysis are available on github: https://github.com/owensgl/sunflower_heterosis_2018. All sequence data are available on the SRA; see supplementary table 1.
