# Supplementary figures and images for: Genomic sequence and copy number evolution during hybrid crop development in sunflowers
Source: Evol Appl. 2018 Feb 20;12(1):54–65. doi: 10.1111/eva.12603 (PMC6304689; doi:10.1111/eva.12603)

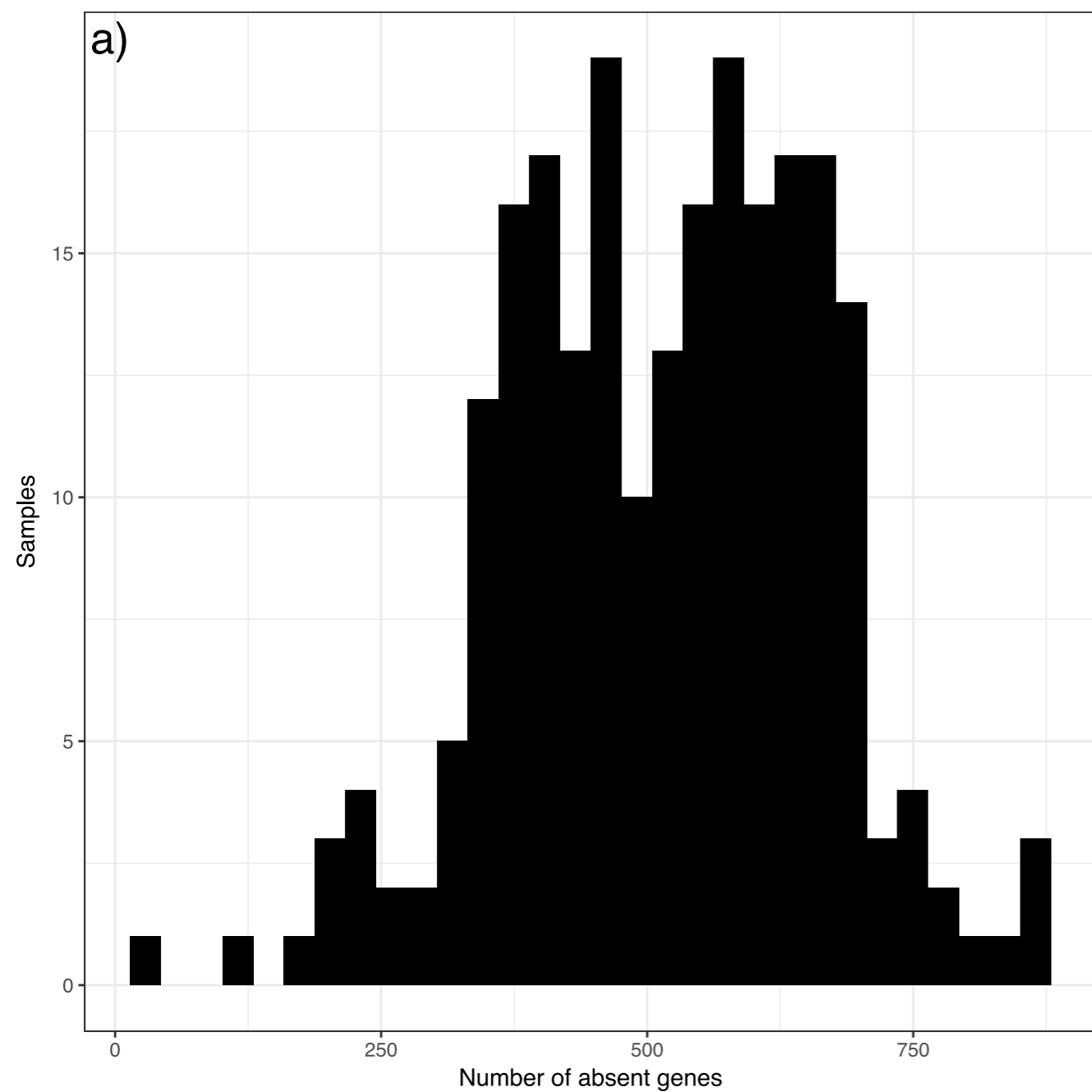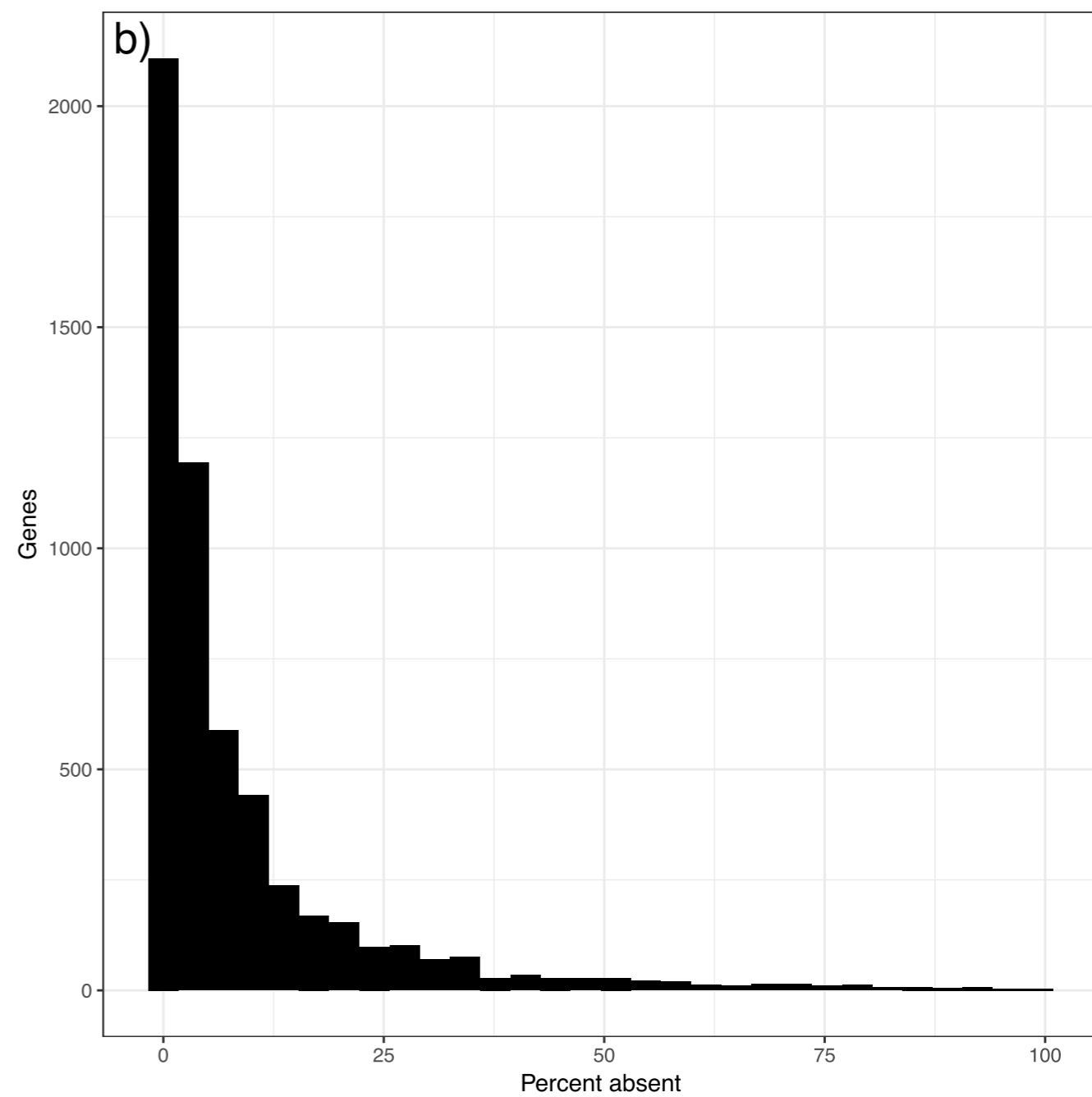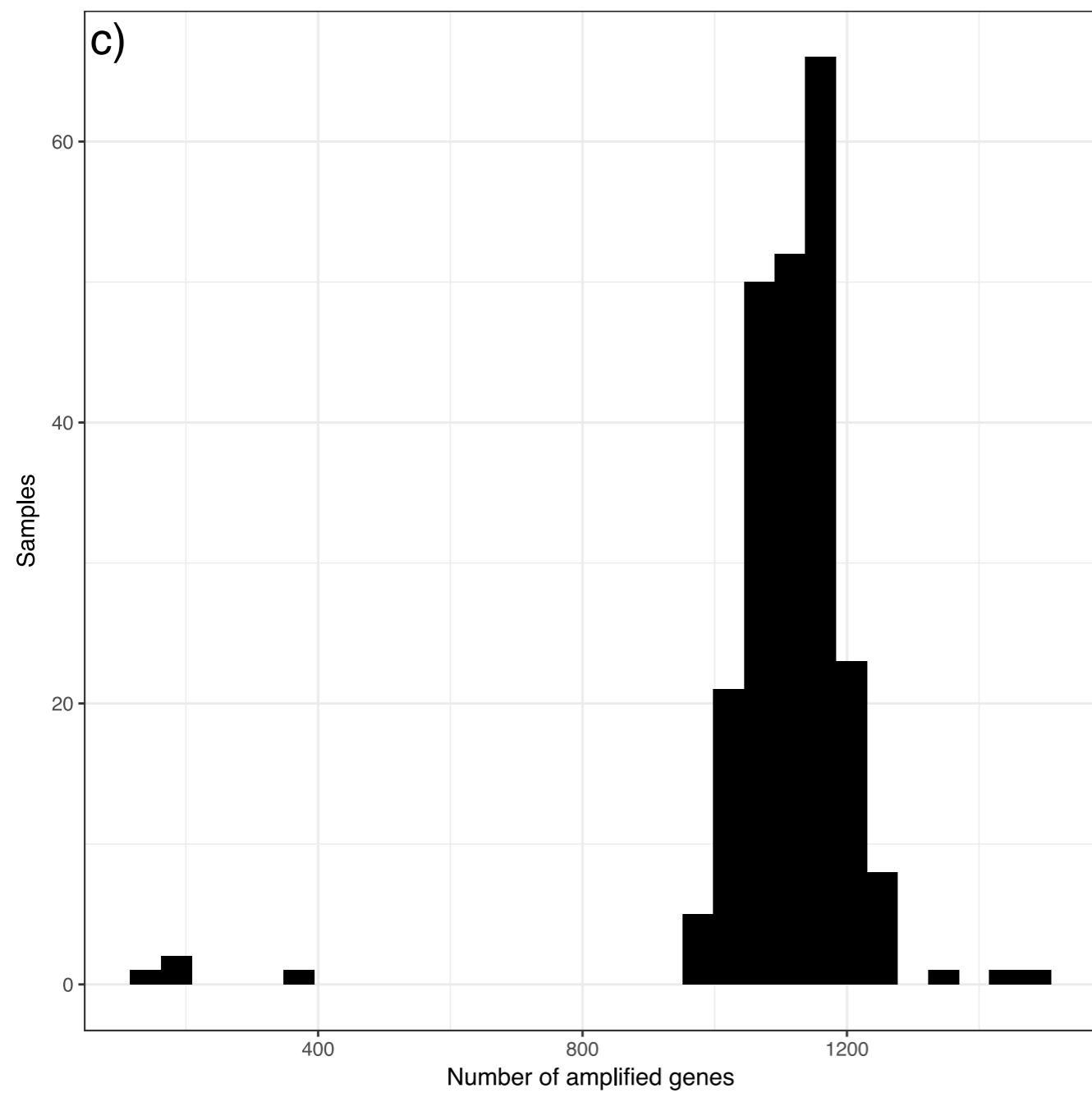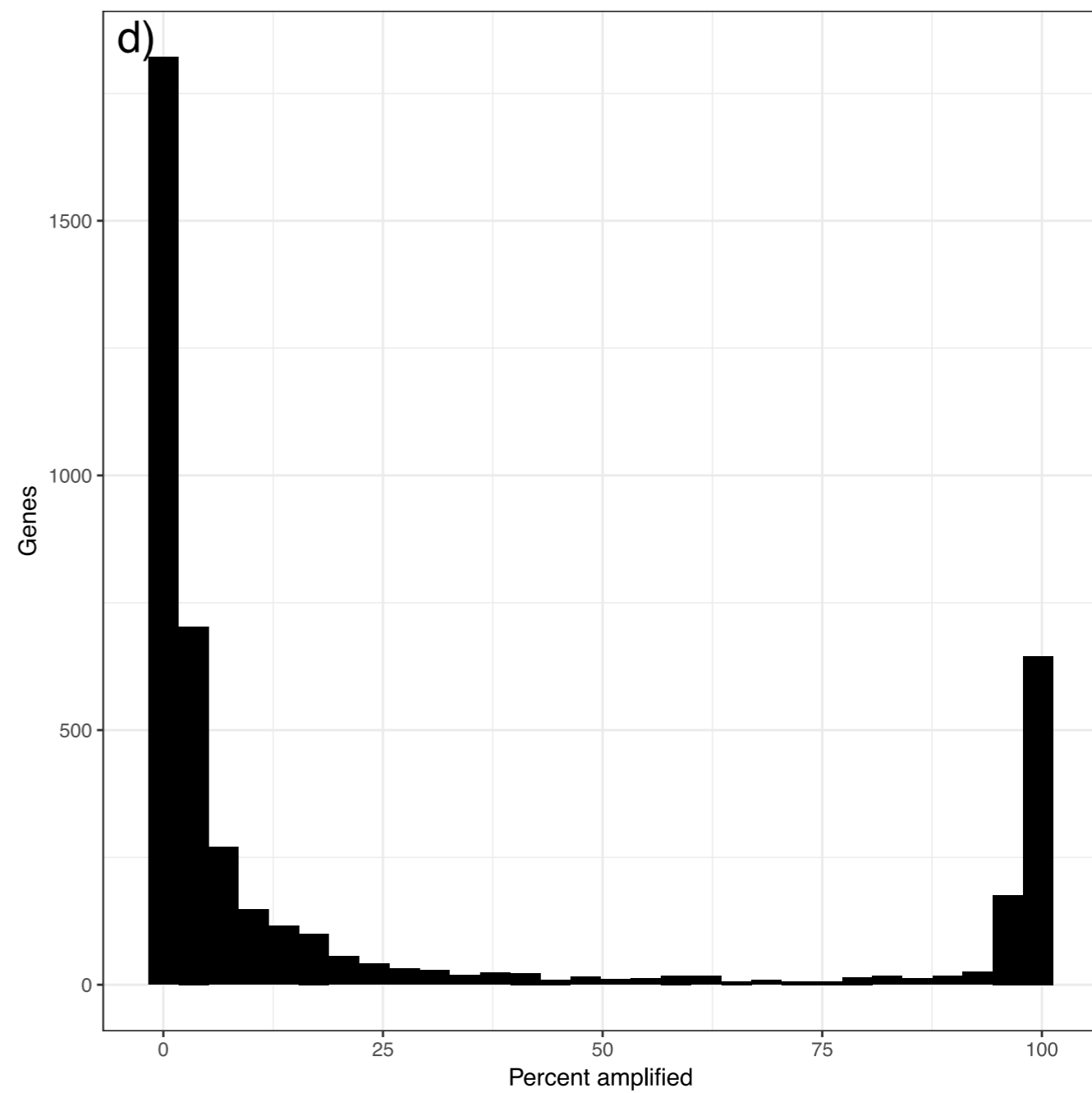

Supplement: Supplementary file 1 [file EVA-12-54-s001.pdf]

B-lines  $f(\text{amplified})$  - R-lines  $f(\text{amplified})$

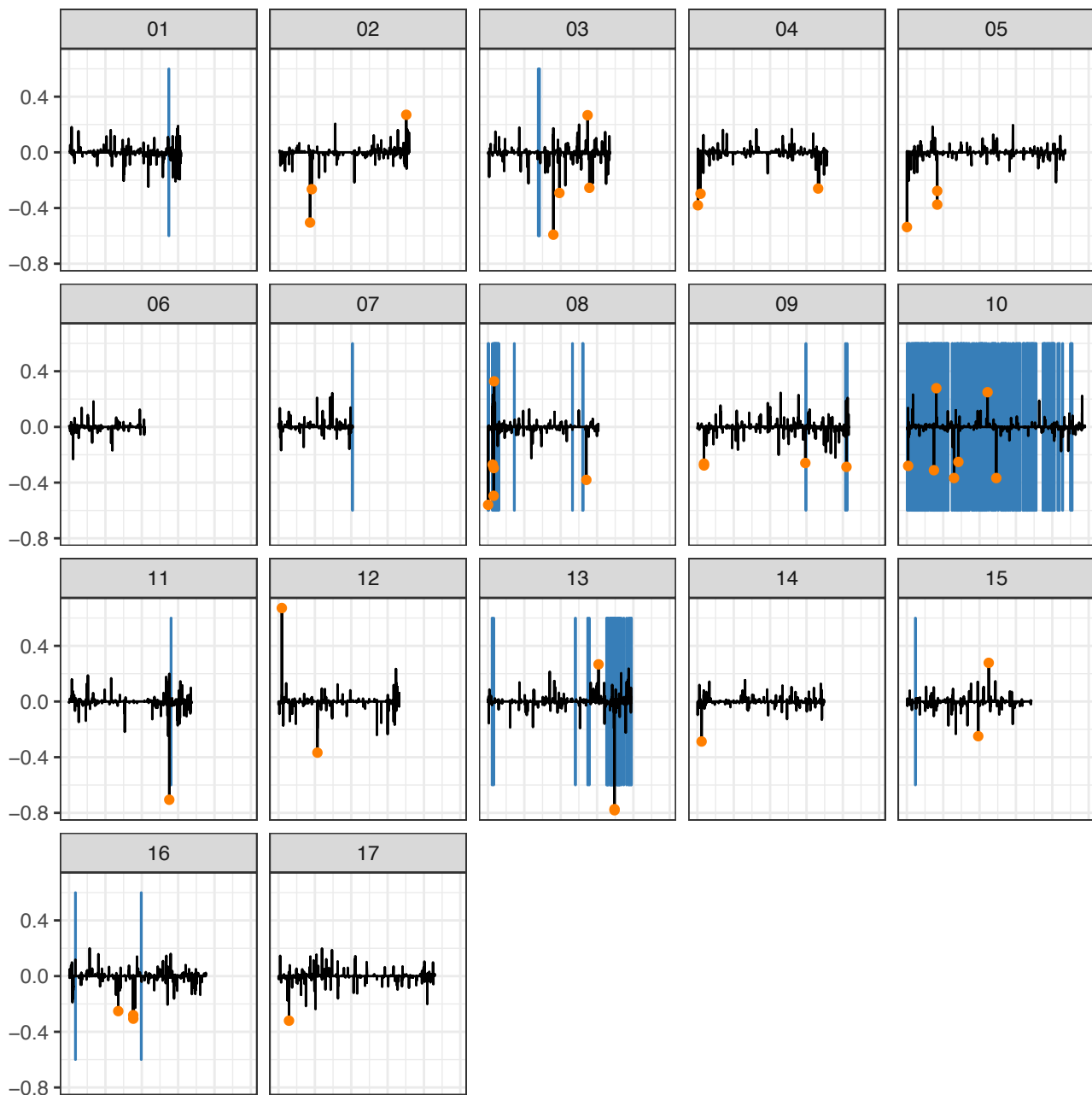

Position

Supplement: Supplementary file 2 [file EVA-12-54-s002.pdf]

# Differentiated P/A genes

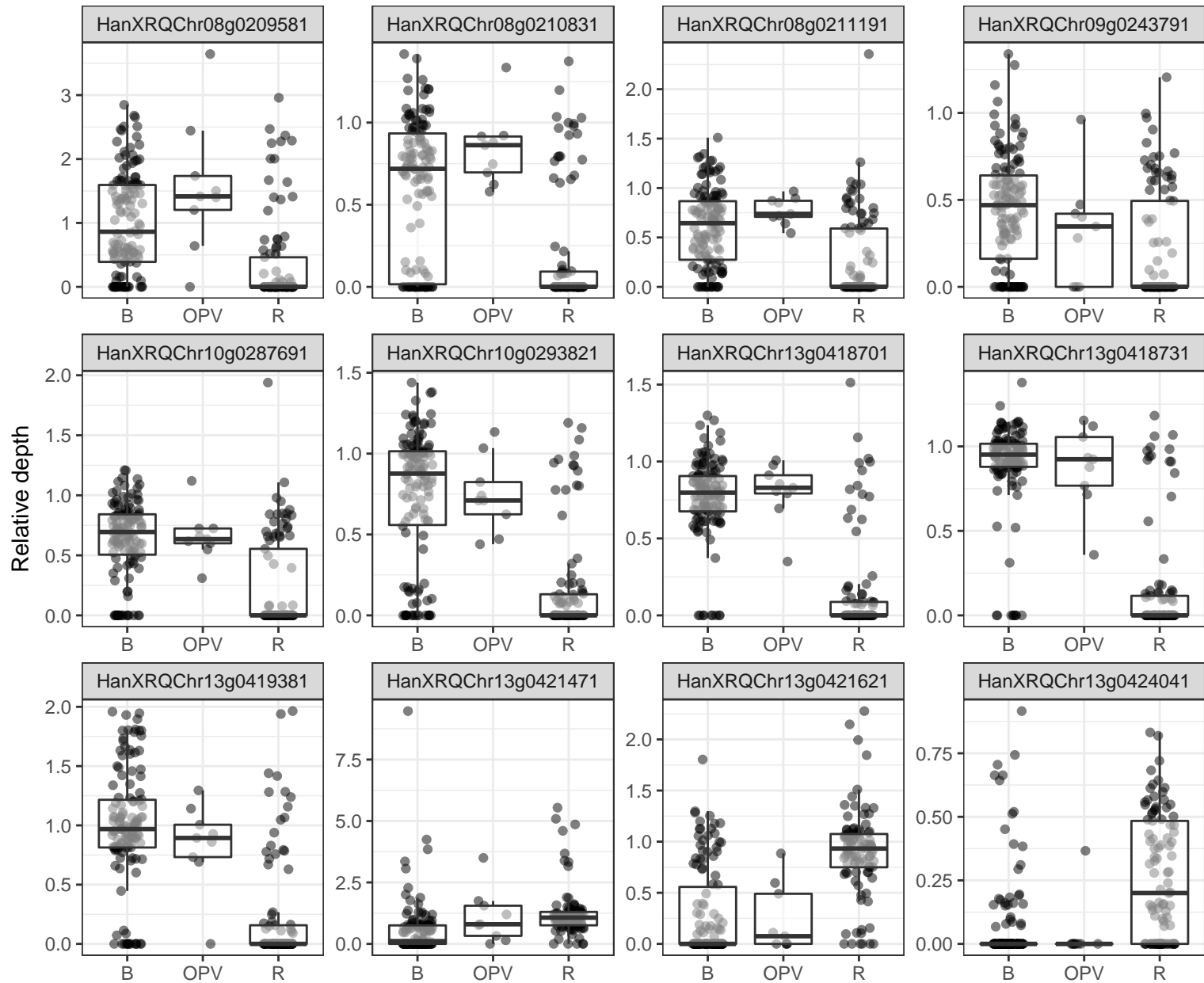

# Differentiated amplified genes

Relative depth

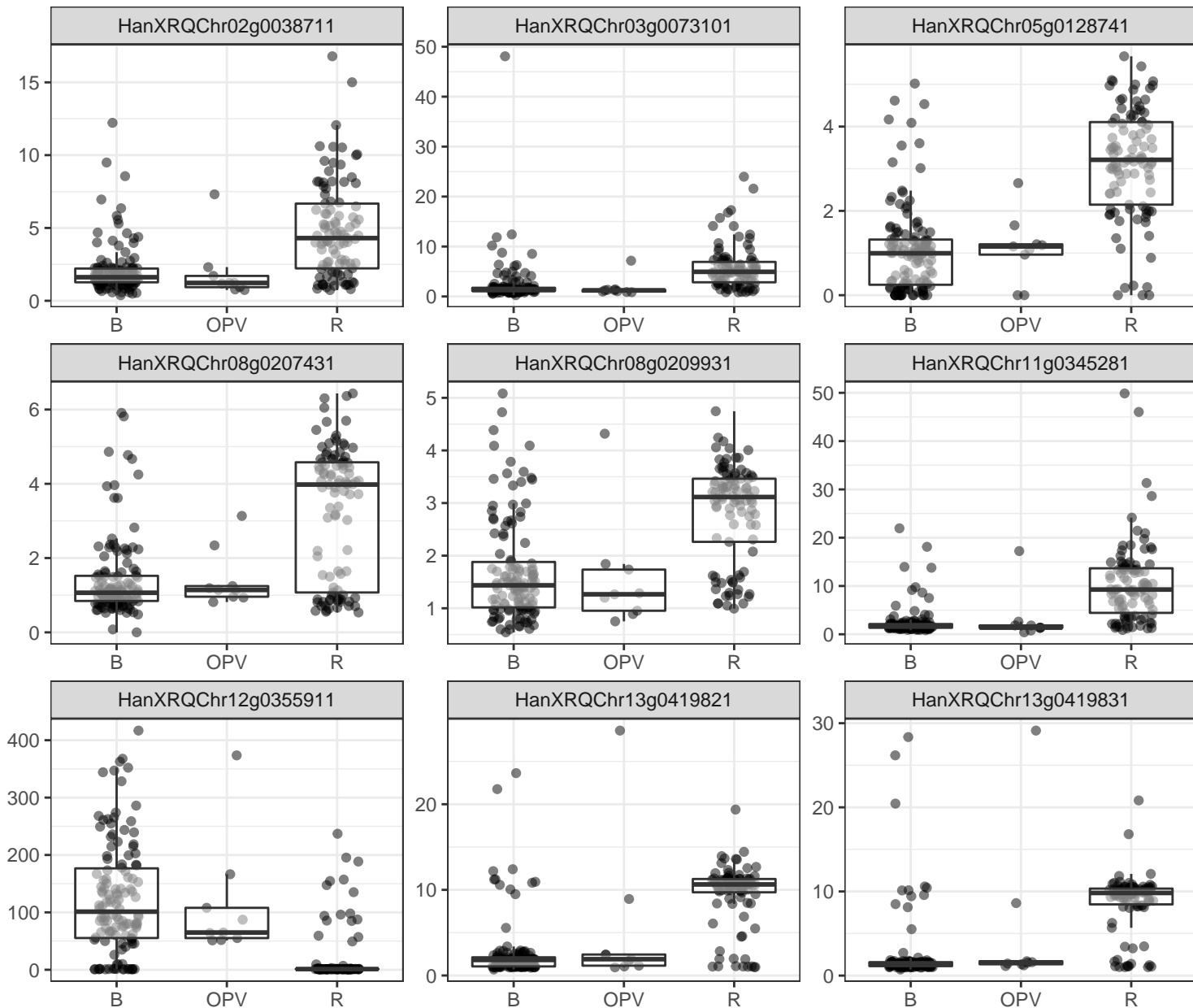

Supplement: Supplementary file 3 [file EVA-12-54-s003.pdf]

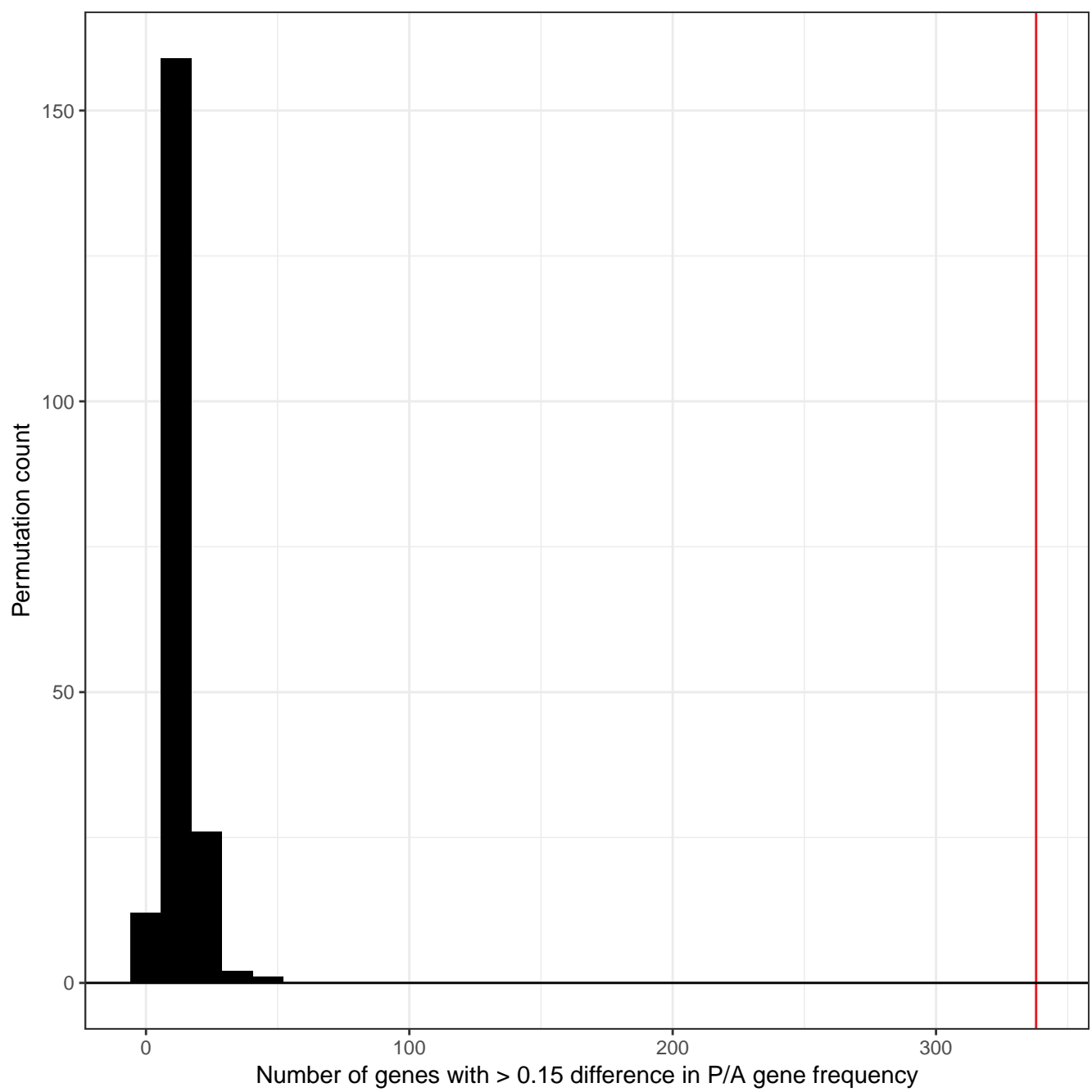

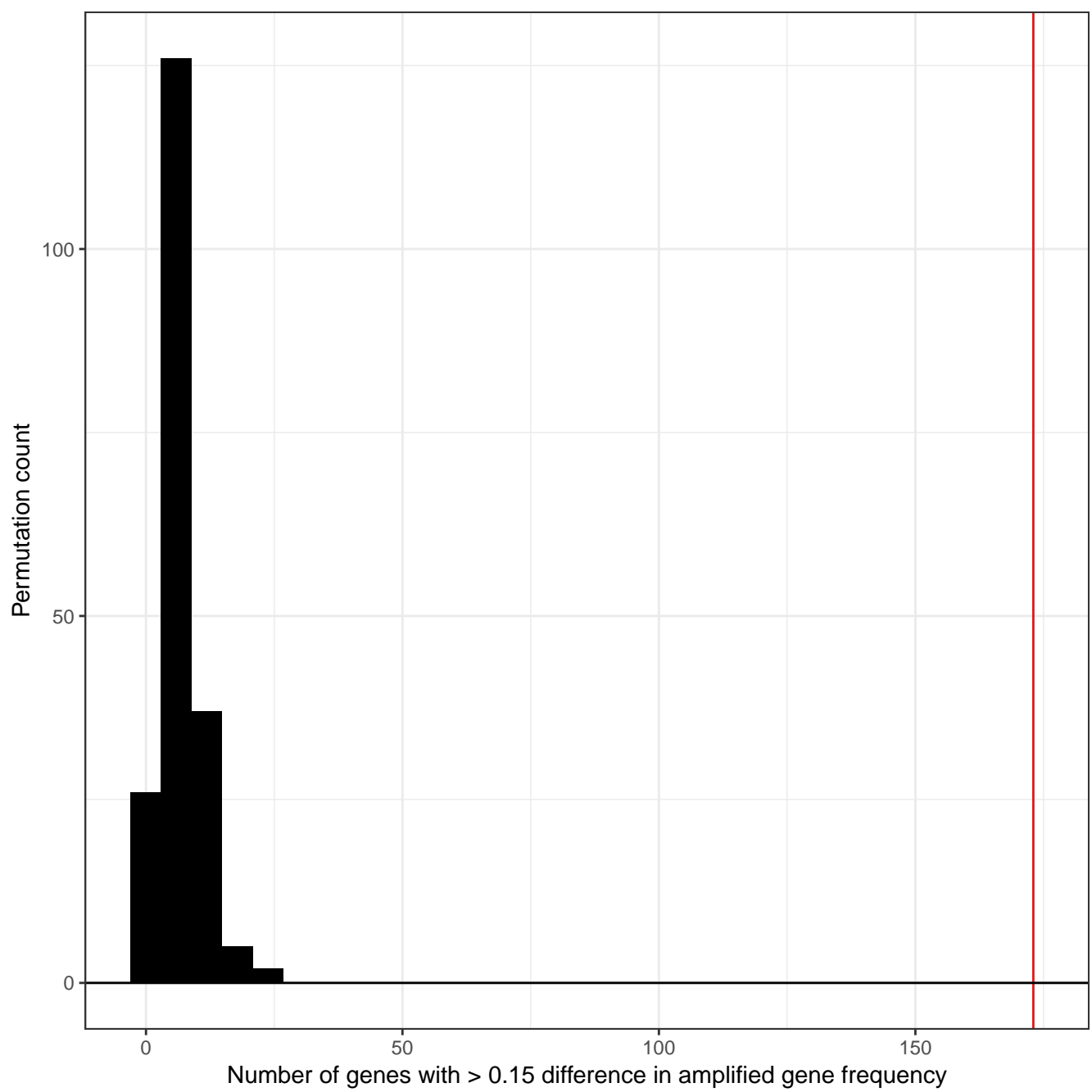

Supplement: Supplementary file 4 [file EVA-12-54-s004.pdf]

Frequency of opposite sorting in B- and R-lines

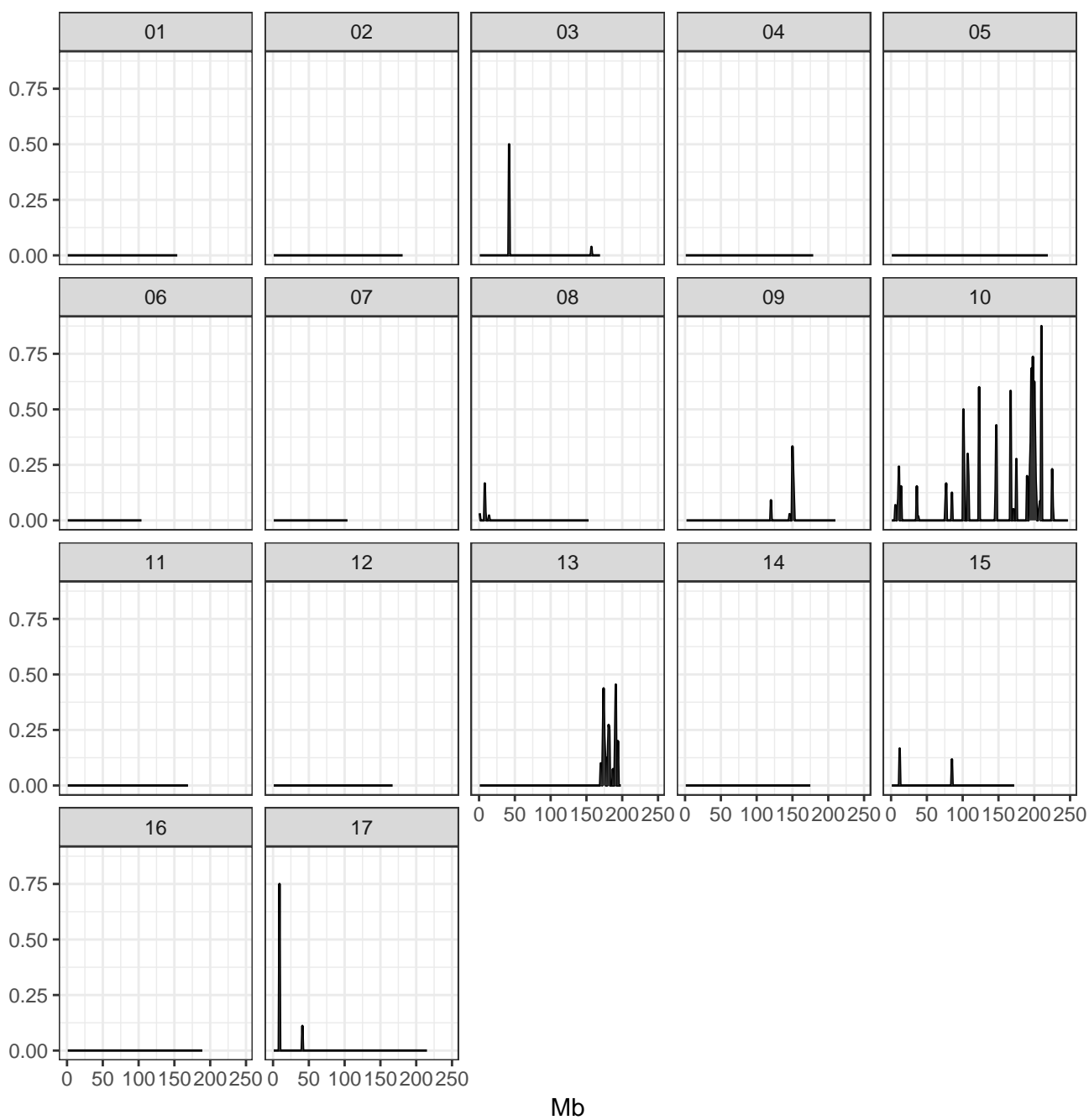

Supplement: Supplementary file 5 [file EVA-12-54-s005.pdf]

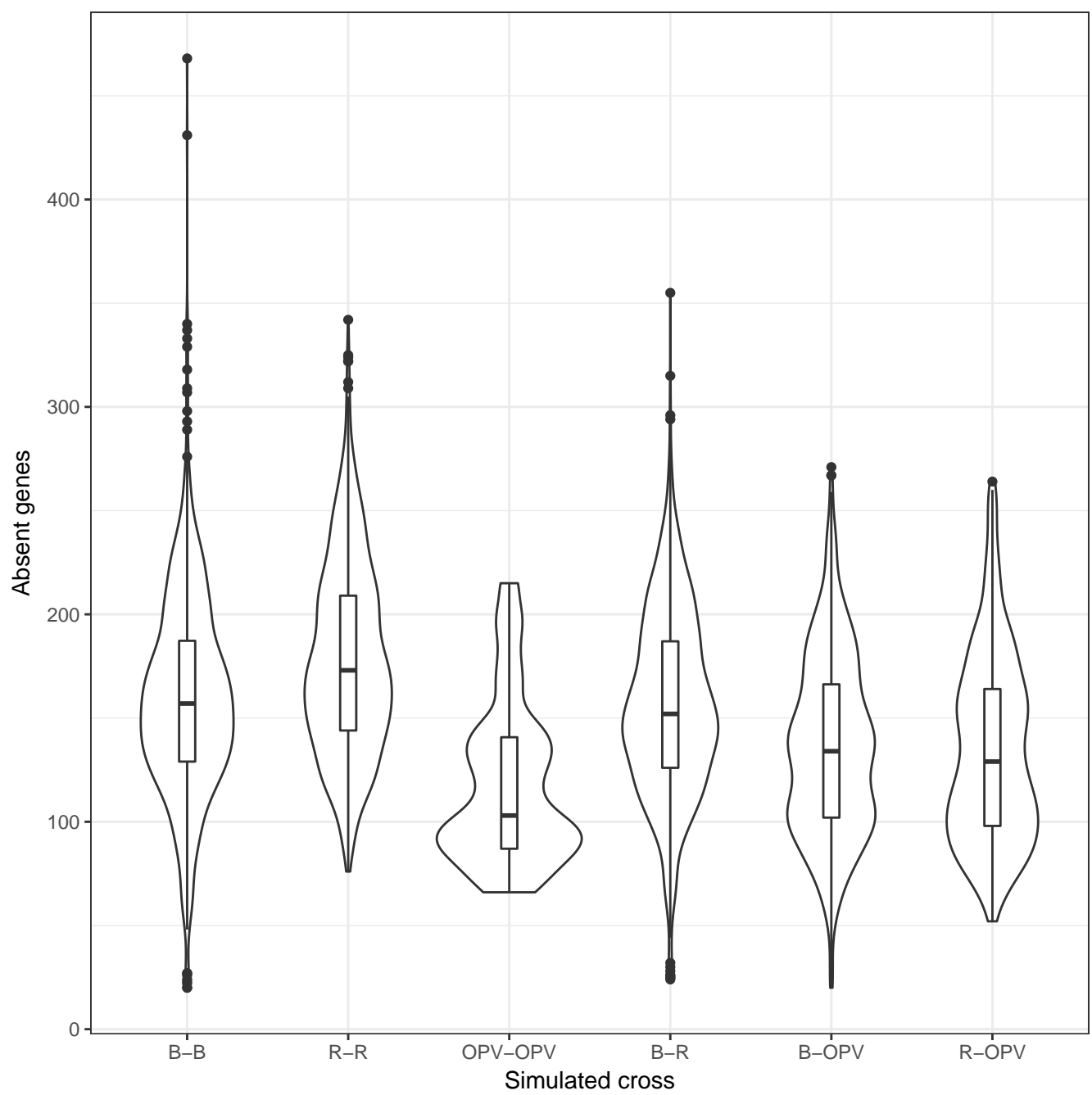

Supplement: Supplementary file 6 [file EVA-12-54-s006.pdf]

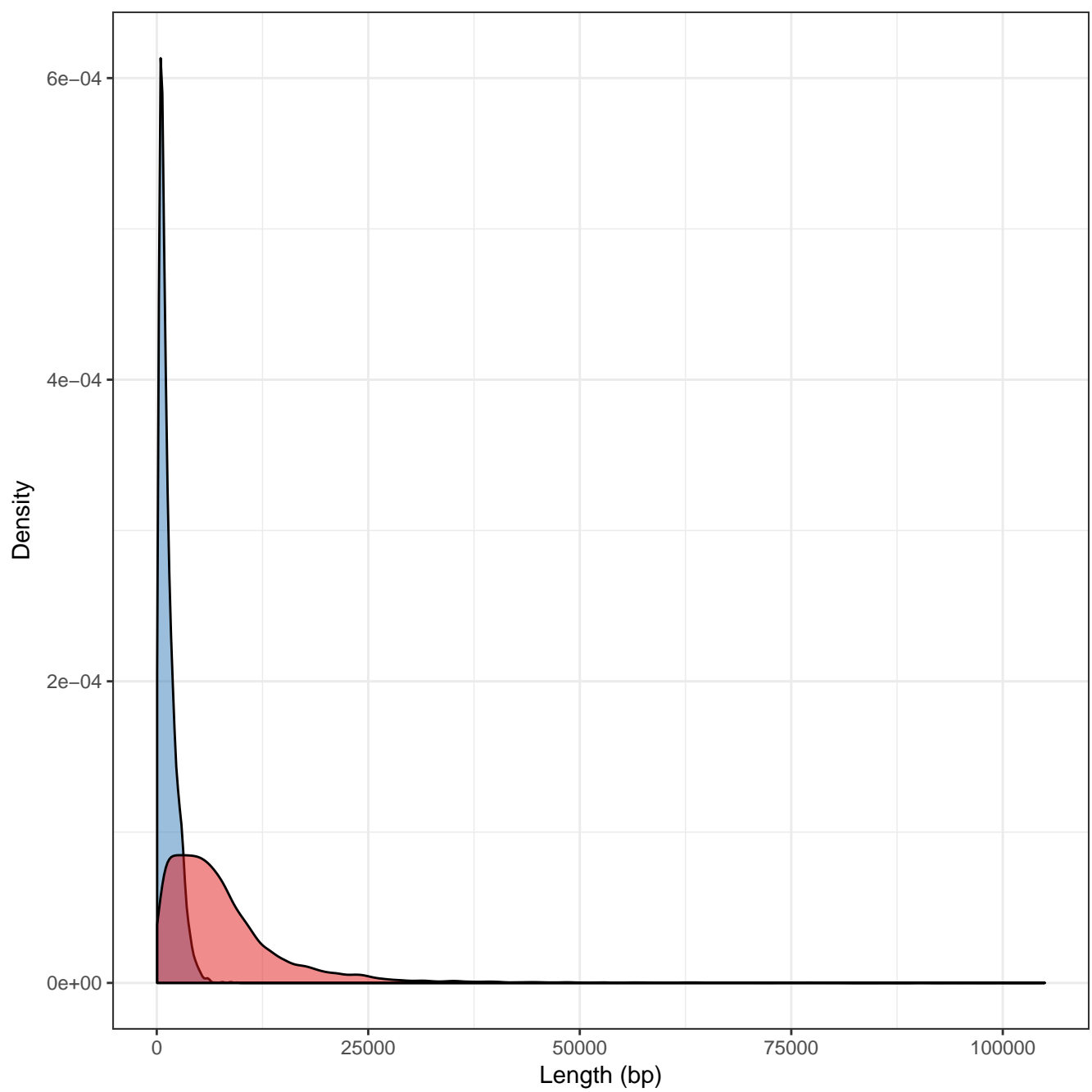

Supplement: Supplementary file 7 [file EVA-12-54-s007.pdf]

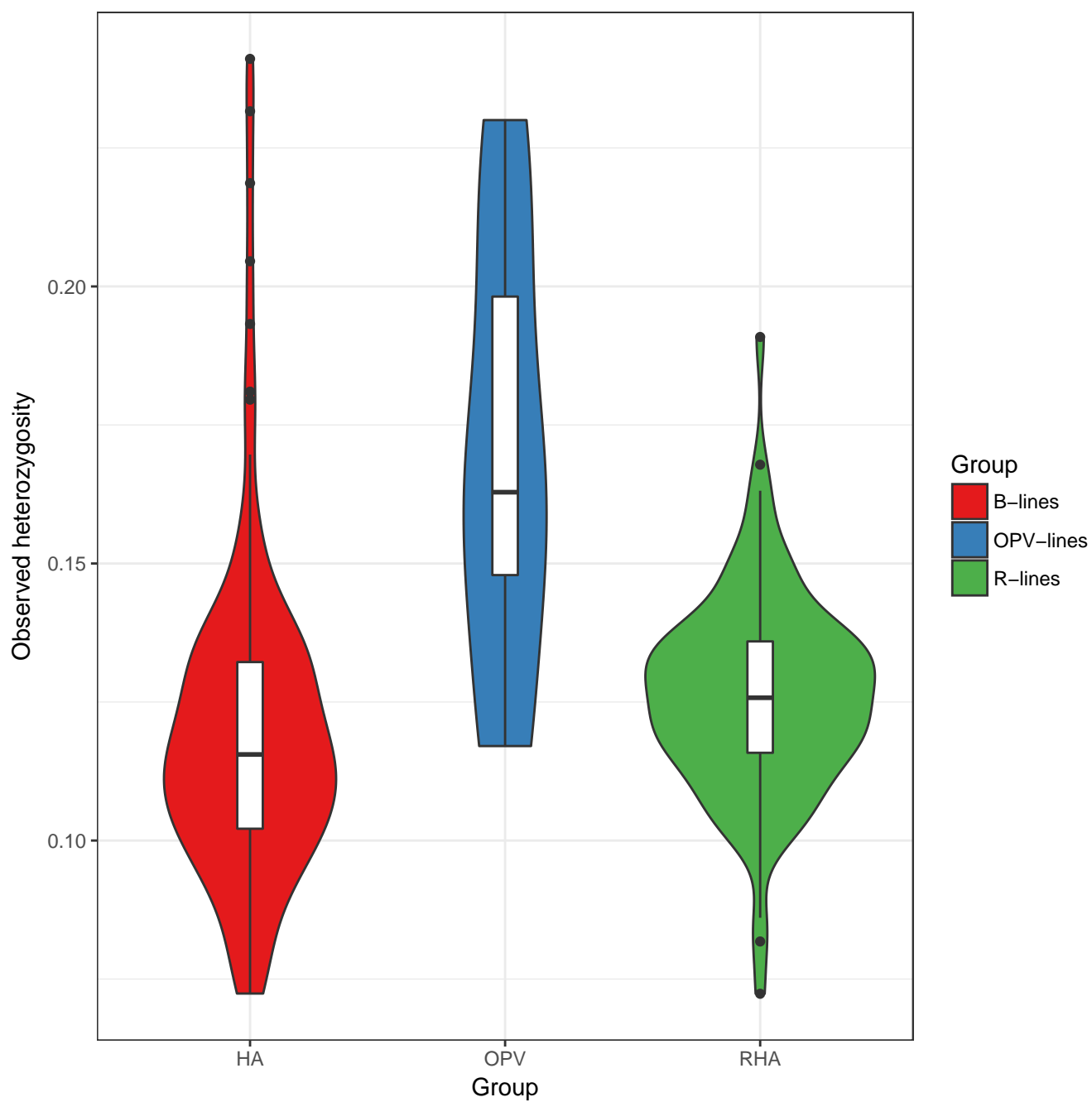

Supplement: Supplementary file 8 [file EVA-12-54-s008.pdf]
